# Pulmonary Kaposi Sarcoma: An Uncommon Cause of Respiratory Failure in the Era of Highly Active Antiretroviral Therapy—Case Report and Review of the Literature

**DOI:** 10.1155/2016/9354136

**Published:** 2016-10-30

**Authors:** Stanley M. Nwabudike, Stefan Hemmings, Yonette Paul, Yordanis Habtegebriel, Octavius Polk, Alem Mehari

**Affiliations:** ^1^Department of Internal Medicine, Howard University College of Medicine, Washington, DC, USA; ^2^Division of Pulmonary and Critical Care, Howard University College of Medicine, Washington, DC, USA

## Abstract

Kaposi Sarcoma (KS) is the most common malignancy associated with Acquired Immune Deficiency Syndrome (AIDS) and is caused by Human Herpesvirus 8 (HHV 8) or Kaposi Sarcoma Herpesvirus (KSHV). In about 90% of cases Kaposi Sarcoma is associated with cutaneous lesions; however visceral disease can occur in the absence of cutaneous involvement. In the era of Highly Active Antiretroviral Therapy (HAART), the incidence of KS has declined. Clinical features of pulmonary KS might be difficult to distinguish from pneumonia in the immunocompromised patients and could lead to diagnostic challenges. First-line treatment of KS is with HAART and the incidence has declined with its use. Systemic chemotherapy may play a role depending on the extent of the disease. We report the case of a young man who presented with pulmonary symptoms and was later found to have pulmonary KS. Interestingly this diagnosis was made in the absence of the classic skin lesions. His disease was complicated by progressive respiratory failure and he eventually died.

## 1. Introduction 

Kaposi Sarcoma is a vascular tumor of the blood vessels and lymph nodes associated with the Human Herpesvirus 8 (HHV-8) also known as the KS Herpesvirus (KSHV) [1, 2]. It is an AIDS defining condition according to the Center for Disease Control and Prevention (CDC) and World Health Organization (WHO) [3, 4]. The incidence of KS ranged from 1,500 to 2,500 cases per 100,000 person-years before the widespread use HAART [5, 6]. However with the adoption of HAART the incidence has declined significantly to less than 500 cases per 100,000 person-years [6]. In the Westminster HIV cohort conducted in the early HAART era (1996–2004), 8% of the 305 KS cases had pulmonary involvement. Patients with pulmonary KS had lower CD4 cell counts and were more likely to be of African origin [7]. Other studies have suggested that pulmonary KS is more likely to present in patients with extensive cutaneous disease [8], which is thought to progress to visceral involvement. In contrast, this case of pulmonary KS was diagnosed on bronchoscopy despite the absence of cutaneous lesions. Our case highlights the importance of considering pulmonary Kaposi as a differential diagnosis when patients with AIDS present with pulmonary symptoms, even in the era of HAART.

## 2. Case Report 

A 26-year-old African American man with AIDS presented with complaints of pleuritic chest pain, exertional shortness of breath, and hemoptysis of 3-week duration. He reported 10-pound weight loss in 6 months, drenching night sweats, and fever. He denied tobacco use but admitted using marijuana in the past. He denied neurological, gastrointestinal, or dermatological symptoms and had no recent travel. He was being treated with dapsone and elvitegravir/cobicistat/emtricitabine/tenofovir but was poorly compliant.

Of note, he was admitted 3 months earlier for similar complaints and was diagnosed with atypical pneumonia. A CT scan of the chest done on that admission showed diffuse fine noncalcified nodular densities in both lungs and small to moderate bilateral axillary, mediastinal, and hilar adenopathy. Bronchoscopy was unrevealing for endobronchial lesions. Bronchoalveolar lavage was negative for* Pneumocystis jirovecii*, acid fast bacilli, or fungal infection.

**Figure 1:**
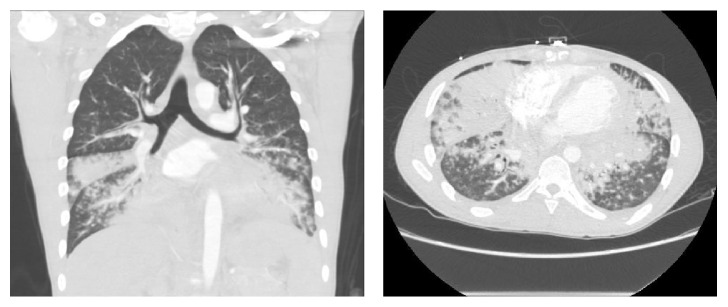
CT of chest with contrast: coronal and axial views showing fibronodular infiltrates with consolidation in the right middle and lower lobes and left upper and lower lobes.

**Figure 2:**
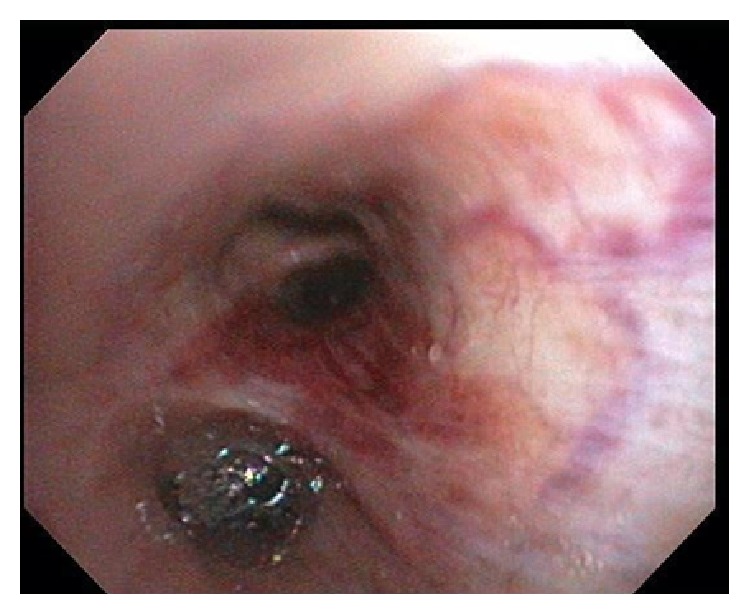
Right lower lobe bronchus showing violaceous raised mucosal lesions.

**Figure 3:**
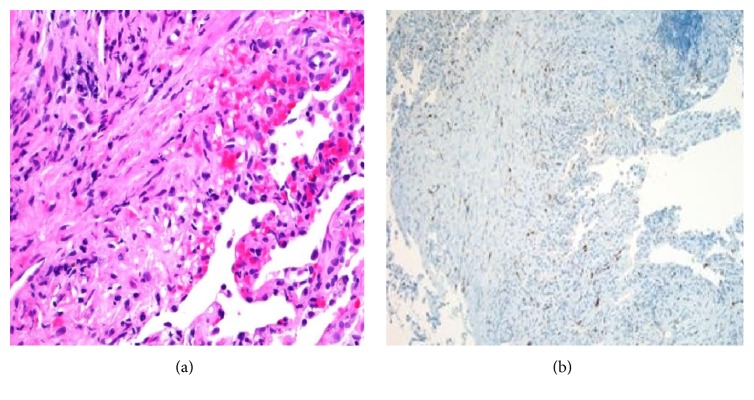
(a) Atypical spindle cell proliferation with slit-like vascular spaces, lymphoplasmacytic infiltrate, and extravasated red blood cells. (b) Nuclear HHV8 immunostain is expressed in the atypical spindle cell proliferation.

On index presentation, he had bilateral axillary and supraclavicular lymphadenopathy with signs of right lower lung zone consolidation on chest examination. There were no lesions on the skin or mucosa. Complete blood count was significant for normocytic anemia and metabolic panel was within normal limits. Absolute CD4 cell count is of 102 cells *μ*/L (17%). A repeat CT scan of the chest showed significant worsening of fibronodular infiltrates with consolidation in the right middle and lower lobes and left upper and lower lobes with bilateral axillary, supraclavicular, mediastinal, and hilar lymphadenopathy (Figure 1). Subsequent bronchoscopy revealed erythematous lesions on the proximal and distal part of the trachea and right middle lobe (Figure 2). Left main stem had vascular markings with dilated veins. These findings were consistent with endobronchial KS. Transbronchial biopsy was done and pathology was positive for atypical spindle cell proliferation with slit-like vascular spaces, lymphoplasmacytic infiltrate, and extravasated red blood cells (Figure 3). Nuclear HHV8 immunostain was expressed in atypical cells consistent with Kaposi Sarcoma.

He was started on a different HAART regimen: abacavir/dolutegravir/lamivudine after consultation with the infectious disease team. The patient tolerated the new medication and was discharged with appropriate follow-up. However he was readmitted twice: once with Immune Reconstitution Syndrome and the second time with massive hemoptysis. He developed acute respiratory failure, which unfortunately led to his death.

## 3. Discussion 

Kaposi Sarcoma is a slowly growing angioproliferative tumor, involving blood vessels and lymph nodes. It was eponymously named after a Hungarian dermatologist, Moritz Kaposi in 1872 after he described this disease in elderly men of Mediterranean origin [9]. There are 4 types: classic, endemic, organ transplant-associated, and epidemic or AIDS related, the latter being the most common malignancy associated with AIDS. Although the disease had been discovered over a century ago, it was not until 1994 that viral etiology was identified. The Kaposi Sarcoma Human Herpesvirus (KSHV/HHV-8) was detected in KS biopsies from AIDS patients [1] and has now been firmly established as the cause of this malignancy. Although HHV8 DNA was first described in skin biopsies of HIV positive patients, it is also found in HIV negative patients with KS. HHV8 DNA can also be found in visceral KS lesions and in the peripheral blood of patients with KS [2, 10]. Subsequent to its discovery in AIDS patients with KS, HHV-8 has also been linked to other conditions: body cavity based lymphoma (also known as primary effusion lymphoma or PEL) and Castleman's disease.

KS is more common in AIDS patients than in the general population and even more so among homosexual and bisexual men [11]. Pulmonary KS however has also been known to occur in HIV negative patients, though uncommon [2, 12]. Prior to the AIDS pandemic, KS was a rare malignancy. KS may also present for the first time as part of the Immune Reconstitution Inflammatory Syndrome (IRIS), with pulmonary involvement occurring more commonly in IRIS-associated KS [13]. Unlike most other AIDS related malignancies, there has been a decreased incidence of KS and improved survival since the advent of HAART [14, 15].

Pulmonary Kaposi Sarcoma is often indistinguishable from other opportunistic lung diseases in patients with AIDS; therefore it is important to have a high index of suspicion to make a diagnosis especially in the absence of cutaneous manifestations. This is even more crucial because treatment with steroids can lead to rapid progression of KS symptoms.

Clinical features oftentimes include nonproductive cough, hemoptysis, weight loss, hypoxemia, and fever. In about 90% of cases these occur with cutaneous involvement [16]. Laboratory findings may include anemia with raised HIV viral load and evidence of profound immunosuppression: CD4 counts are usually less than 200 [16, 17]. A lower CD4 count has been shown to proffer a poorer prognosis [18]. Imaging findings include reticulonodular opacities described as flame shaped lesions that may occur along the lines of bronchovascular bundles, perihilar infiltrates pleural effusion, and hilar adenopathy on radiographs or CT scan. These may again be difficult to distinguish from other pulmonary pathology on chest imaging, especially in a patient with AIDS. Serum LDH may help to further distinguish pulmonary KS from other opportunistic pulmonary disease. In a study by Huang et al., among HIV patients with AIDS related Kaposi Sarcoma, those with concurrent opportunistic pneumonia had a higher median LDH than those with pulmonary KS alone [16].

The findings on bronchoscopy are pathognomonic and typically appear as violaceous or bright red maculopapular lesions of the mucosa of the lower airways and less commonly in the trachea. Occasionally this classic finding might be absent and the only bronchoscopic evidence might be hyperemia, edema of the lower airways, or complete occlusion of the endobronchial tree [19, 20]. Biopsy may be challenging due to the highly vascular nature of the malignancy with associated increased risk of bleeding and in such cases PCR for HHV-8 can be done on the bronchoalveolar lavage sample [19]. Detection of HHV-8 can be as high as 80% in BAL from HIV positive patients with endobronchial KS [21]. In retrospect, our patient may have had the benefit of an earlier diagnosis if PCR for HHV-8 was done on the BAL on his first admission.

The corner stone of treatment is antiretroviral therapy. This is validated by the significant difference in outcomes since the era of HAART [14, 15, 22], with decreased incidence and improved survival. There is a risk of Immune Reconstitution Inflammatory Syndrome after starting HAART for treatment of KS, as was seen in our patient. This is more likely to occur in patients with a relatively higher CD4 count at the initiation of HAART [23]. Systemic chemotherapy with pegylated liposomal anthracyclines, immunotherapy with IFN-alpha, or thalidomide can be used especially when there is evidence of advanced disease or risk of disease progression. Other considerations which may prompt concomitant use of HAART and systemic chemotherapy include symptomatic visceral disease or cytoreduction in cases of large bulky tumors. Significant risk of toxicities from chemotherapy may however limit its use [24, 25]. For cosmetic reasons intralesional chemotherapy, radiation therapy, and topical retinoids may be used.

## 4. Conclusion 

Pulmonary KS being a common malignancy associated with AIDS should be suspected in patients who present with respiratory failure despite negative bronchoscopic finding and the absence of cutaneous disease. It is a severe manifestation of KS disease and has been associated with poor outcomes. A high index of suspicion is required for prompt diagnosis and treatment. Bronchoscopy and biopsy or BAL with PCR testing for HHV-8 can be done in suspected cases. When KS is diagnosed, HAART is the first line for treatment and its use has been shown to markedly improve the prognosis of this disease.
